# The complete mitochondrial genome of the larval Bombay duck *Harpodon nehereus* (Aulopiformes, Synodontidae) from Yangtze estuary and the phylogenetic relationship of Synodontidae species

**DOI:** 10.1080/23802359.2018.1476070

**Published:** 2018-05-26

**Authors:** Hui Zhang, Weiwei Xian

**Affiliations:** a CAS Key Laboratory of Marine Ecology and Environmental Sciences, Institute of Oceanology, Chinese Academy of Sciences, Qingdao, China;; b Laboratory for Marine Ecology and Environmental Science, Qingdao National Laboratory for Marine Science and Technology, Qingdao, China

**Keywords:** *Harpodon nehereus*, larvae fish, mitochondrial genome, next generation sequencing

## Abstract

The complete mitochondrial genome of the larvae *Harpodon nehereus* collected from Yangtze estuary was determined by next-generation sequencing. The mitogenome is a circular molecule 16,536 bp in length, including the typical structure of 13 protein-coding genes, two ribosomal RNA genes, 22 transfer RNA genes, and a control region. The TAS, central CSB and CSB were detected in the control region. The gene contents of the mitogenome are identical to those observed in most marine bony fishes. The NJ tree showed that *H. nehereus* clustered in a branch close to the species from the same genus.

*Harpodon nehereus* with the common name Bombay duck distributes extensively in the Pacific Ocean and Indian Ocean and is caught off the eastern coast of China from March to May and also from August to November (Jin et al. 2012). The annual catch of *H. nehereus* in China is more than 300,000 metric tons. So it is a commercially important fish. Next generation sequencing (NGS) technologies provide an effective platform for the development of genetic markers, such as the mitochondrial genome that can be used to provide insight into population processes and the evolutionary history of species (Schuster 2008). By exploiting certain tissue types, such as muscle, total genomic DNA extractions can contain high concentrations of mitochondrial DNA (mtDNA), which may then be overrepresented in NGS analyses (Dalziel et al. 2005). In the present study, we use NGS by extracting DNA from muscle tissue of the larvae *H. nehereus* collected from Yangtze estuary (E 122.33°, N 31.25°) in the November 2016 and undertaking a modest 454 NGS analysis to isolate mtDNA sequences. The DNA is stored at Fisheries Group, CAS Key Laboratory of Marine Ecology and Environmental Sciences with the no. 2016-11-D01.

Complete mitogenome of *H. nehereus* was 16,536 bp in length (GenBank accession no. MH204885). As in other vertebrates (Miya et al. 2001), it contained 13 protein-coding genes, two rRNA genes (12S rRNA and 16S rRNA), 22 tRNA genes, and a control region. Like other bony fishes, most mitochondrial genes of *H. nehereus* were encoded on the H-strand, with only ND6 and eight tRNA (Gln, Ala, Asn, Cys, Tyr, Ser-UCN, Glu, and Pro) genes encoded on the L-strand. Among 13 protein coding genes, two overlapping reading frames were detected on the same strand. The ATPase 6 and ATPase 8 overlap by 10 nucleotides, and ND4 and ND4L share seven nucleotides. ND5 and ND6 overlap by four nucleotides on the opposite strand. ATG is the initiation codon of 12 out of the 13 protein coding genes (ND1, ND2, CO2, ATPase 8, ATPase 6, CO3, ND3, ND4L, ND4, ND5, ND6, and Cyt b), while the initiation codon of COI is GTG. TAA is the stop codon for five genes (ND1, COI, ATPase 8, ND4L, and ND5), TAG is the stop codon for ND6, and the other genes have incomplete stop codons TA or T-, which are presumably completed as TAA by post-transcriptional polyadenylation (Ojala et al. 1981). The 12S and 16S ribosomal RNA genes of *H. nehereus* comprise 946 bp and 1658 bp, respectively. They are located between tRNA^Phe^ and tRNA^Leu^ (UUR) as they are in other vertebrates (Zhang and Xian 2016). The 22 tRNA genes are interspersed in the genome and range in size from 65 to 73 bp and fold into cloverleaf secondary structures with normal base paring. The control region in *H. nehereus* is located between tRNA^Pro^ and tRNA^Phe^, and was determined to be 911 bp in length. The TAS, central CSB and CSB were detected in the control region, which is similar to most bony fishes (Zhang et al. 2013). Phylogenetic relationship revealed NJ tree among six Synodontidae species based on 12 H-strand mitochondrial protein-coding genes, 22 tRNA, and two rRNA genes. The NJ phylogenetic tree showed that *H. nehereus* clustered in a branch close to the species from the same genus (Figure 1).

**Figure 1.**
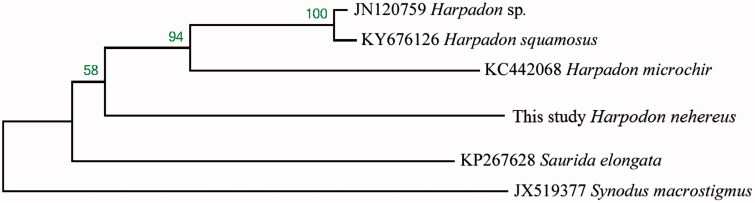
Phylogenetic relationship revealed by NJ tree among six Synodontidae species.
